# Galectin-9 Controls CD40 Signaling through a Tim-3 Independent Mechanism and Redirects the Cytokine Profile of Pathogenic T Cells in Autoimmunity

**DOI:** 10.1371/journal.pone.0038708

**Published:** 2012-06-07

**Authors:** Gisela M. Vaitaitis, David H. Wagner

**Affiliations:** Department of Medicine and Webb-Waring Center, University of Colorado Denver, Aurora, Colorado, United States of America; Johannes Gutenberg University of Mainz, Germany

## Abstract

While it has long been understood that CD40 plays a critical role in the etiology of autoimmunity, glycobiology is emerging as an important contributor. CD40 signaling is also gaining further interest in transplantation and cancer therapies. Work on CD40 signaling has focused on signaling outcomes and blocking of its ligand, CD154, while little is known about the actual receptor itself and its control. We demonstrated that CD40 is in fact several receptors occurring as constellations of differentially glycosylated forms of the protein that can sometimes form hybrid receptors with other proteins. An enticing area of autoimmunity is differential glycosylation of immune molecules leading to altered signaling. Galectins interact with carbohydrates on proteins to effect such signaling alterations. Studying autoimmune prone NOD and non-autoimmune BALB/c mice, here we reveal that *in-vivo* CD40 signals alter the glycosylation status of non-autoimmune derived CD4 T cells to resemble that of autoimmune derived CD4 T cells. Galectin-9 interacts with CD40 and, at higher concentrations, prevents CD40 induced proliferative responses of CD4^lo^CD40^+^ effector T cells and induces cell death through a Tim-3 independent mechanism. Interestingly, galectin-9, at lower concentrations, alters the surface expression of CD3, CD4, and TCR, regulating access to those molecules and thereby redirects the inflammatory cytokine phenotype and CD3 induced proliferation of autoimmune CD4^lo^CD40^+^ T cells. Understanding the dynamics of the CD40 receptor(s) and the impact of glycosylation status in immunity will gain insight into how to maintain useful CD40 signals while shutting down detrimental ones.

## Introduction

It has long been understood that CD40 – CD154 interactions are critical in the etiology and perpetuation of autoimmunity and CD40 is gaining further interest in cancer [1], [2], [3] and tissue transplantation [4], [5] therapies. CD40 (tnfrsf5) belongs to the TNF-receptor super family and like other family members it multimerizes to form the functional receptor [6] which interacts with its natural ligand CD154. CD40 expression was long associated only with antigen presenting cells but in actuality its expression is quite widespread and includes neural [7], endothelial, epithelial [8], adipocyte [9] and T cells [10], [11], [12]. We have identified a CD4^+^ subset of T cells that expresses CD40, CD4^lo^CD40^+^ T cells. Those cells exhibit low surface expression of CD4, CD3 and TCR but have high levels of those molecules intracellularly [13]. In the autoimmune model of Type 1 Diabetes (T1D), the CD4^lo^CD40^+^ T cells expand with progressive insulitis, prior to overt diabetes, and CD4^lo^CD40^+^ T cells are necessary and sufficient in transferring disease [11], [12], [14], [15], [16].

We recently demonstrated that the CD40 receptor is dynamic, consisting of different glycoforms of CD40 isoform I and that the CD40 glycoform profile is different in CD4^lo^CD40^+^ T cells originating from autoimmune conditions compared to the same cells from non-autoimmune conditions [17]. A less glycosylated form of CD40 isoform I is associated with survival and proliferation and CD40 can form hybrid receptors with TNF-receptors (TNFR) 1 and 2 [17] that are not responsive to TNFα but are responsive to crosslinking of TNFR1 and/or 2 by means of antibodies. That crosslinking prevents CD40-induced proliferation but does not kill the cells [17].

An important discovery was that expansion of the CD4^lo^CD40^+^ T cell subset [14] and diabetes onset [18] in the non-obese diabetic (NOD) T1D mouse model is prevented by blocking CD40 – CD154 interaction. This treatment also alters the CD4^lo^CD40^+^ T cell CD40 glycoform profile to resemble that of non-autoimmune animals [17]. Conversely, when non-autoimmune BALB/c mice are CD40 stimulated *in-vivo*, the CD4^lo^CD40^+^ T cell population is expanded and the CD40 glycoform profile becomes similar to that of autoimmune CD4^lo^CD40^+^ T cells [17]. Therefore, while it is known how to prevent expansion of CD4^lo^CD40^+^ T cells there are no known mechanisms to control CD4^lo^CD40^+^ T cells once they are expanded.

Glycosylation impacts immunological functions including cell trafficking, regulation of TCR activation threshold, and T cell polarization into Th1, Th2 or Th17 by altering the molecular interactions of immune molecules at the cell surface [19]. Glycosylation deficiency has been associated with autoimmune disease in mice [20], [21], [22] and autoimmune prone mouse strains, including NOD, have been shown to express 30–40% less β1,6GlcNAc-branched N-glycans than non-autoimmune prone strains [23]. A reduction of GlcNAc-branching by 20–25% enhances TCR signaling and T cell proliferation [20].

Galectins are a subgroup of lectins that are defined by their ability to bind β-galactosides in the carbohydrates of glycoproteins [24]. Several galectins regulate T cell function, for example galectin-3 interacts with carbohydrates on TCR molecules, limiting TCR clustering in response to stimulus [22]. In glycosylation-deficient mice that interaction is disrupted, resulting in lowered TCR activation threshold [22]. Galectin-3 can also prevent apoptosis and increase proliferation of T cells [25]. Other examples include galectin-1 which binds the ganglioside GM-1 in effector T-cells causing autoimmune suppression [26] and galectin-9 which attenuates Th1 responses [27] and is protective against diabetes in the NOD T1D mouse model [28]. It was shown that galectin-9 exerts its effect on Th1 cells via interaction with T-cell immunoglobulin and mucin domain-containing protein 3 (Tim-3) [27], however, it was recently demonstrated that galectin-9 also regulates T helper function independently of that molecule [29].

Because galectin-9 controlled Th1 effector cells [27] we determined whether it could also control the pathogenic CD4^lo^CD40^+^ T cells in autoimmunity.

Here we demonstrate that galectin-9 can control CD40 signaling in NOD CD4^lo^CD40^+^ T cells, even 24 hours after signaling was initiated and, if present in higher concentrations, it induces Tim-3 independent cell death in those cells. We demonstrate that expanded CD4^lo^CD40^+^ T cells from BALB/c mice are able to proliferate in response to CD40 signals *in-vitro* while non-expanded CD4^lo^CD40^+^ T cells from control BALB/c mice are not [13]. Galectin-9 also controls CD40 induced proliferation in those expanded BALB/c CD4^lo^CD40^+^ T cells. Interestingly, CD40 signals alter the glycosylation status of non-autoimmune CD4 T cells to appear more like that of autoimmune CD4 T cells. At lower, sub-lethal concentrations, galectin-9 causes up-regulation of CD3, CD4, TCR and CD5 on the surface of the autoimmune CD4^lo^CD40^+^ T cells. The up-regulation of CD3 on the surface renders the cells more responsive to CD3, increasing CD3 induced proliferation. Sub-lethal galectin-9 concentrations also alter the cytokine production by these cells in response to both CD3 and CD40 signals, decreasing CD40 induced IFNγ and IL-6 and increasing CD3 induced IL-2 suggesting that the presence/absence of galectin-9 may govern the pathogenicity of CD4^lo^CD40^+^ T cells.

A greater understanding of the dynamics of CD40 and CD3 signaling and the impact of the availability of those molecules for signaling on the surface of the cells will be imperative in understanding how to control those cells in autoimmunity.

## Results

### Galectin-9 prevents CD40 induced survival and proliferation

**Figure 1:**
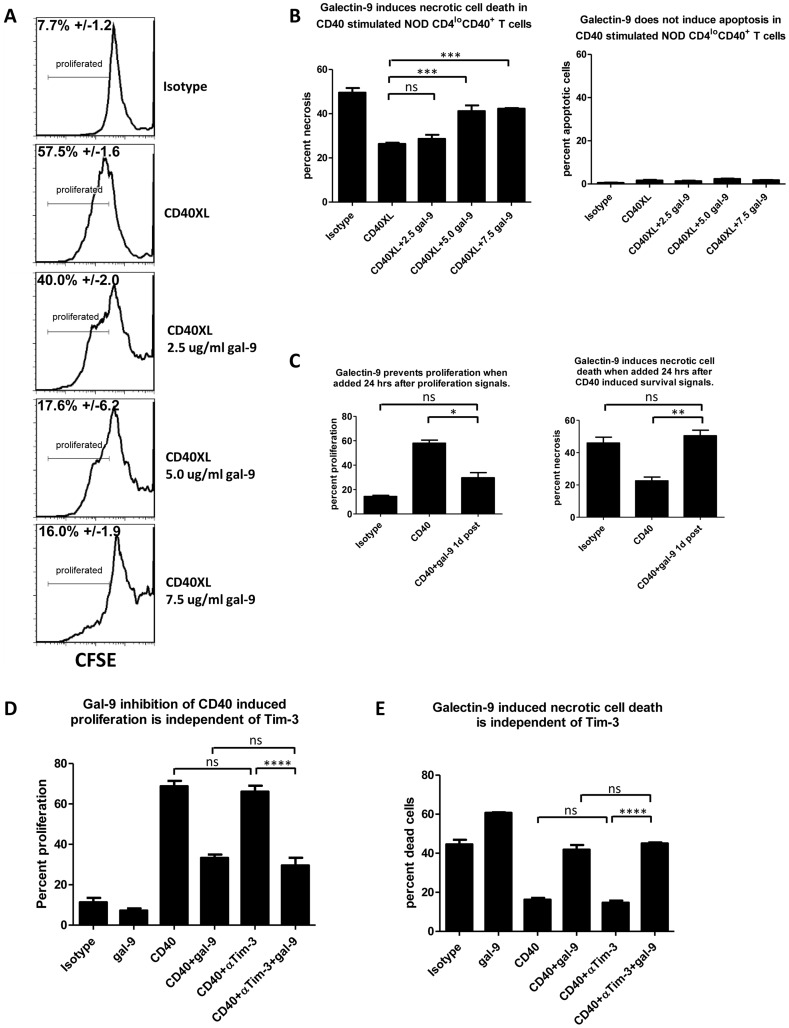
Galectin-9 prevents CD40 induced proliferation. (**A**) CD4^lo^CD40^+^ T cells were sorted from 7–13 week old female NOD spleens and were then labeled with CFSE. Cells were either isotype treated (Isotype) or CD40 was stimulated (CD40XL) in the absence/presence of increasing concentrations of galectin-9 (gal-9) for 4 days then CFSE dilution was measured. (**B**) Cells were sorted and treated as in A and were then stained for necrotic and apoptotic cell death. (**C**) Cells were sorted and CD40 stimulated as in A for 24 hours then galectin-9 was added at 7.5 µg/ml. Cells were analyzed for proliferation and cell death after a total of 4 days of stimulation. (**D and E**) Cells were sorted as in A and were pretreated, or not, with a Tim-3 blocking antibody (αTim-3) for 30 miutes then CD40 was stimulated in the absence/presence of galectin-9 for 4 days. Proliferation (D) and cell death (E) was measured, respectively. Percentages in A are means with SEM. Asterisks in B, C, and D denote significant differences determined by two-way Anova; ns – not significant; * – P between 0.01 and 0.05; ** – P <0.01; *** – P <0.001; **** – P<0.0001. Experiments were done at least three separate times.

Expansion of CD4^lo^CD40^+^ T cells can be prevented by blocking CD40 – CD154 interactions [14], [17]. To date little is known about how to control CD4^lo^CD40^+^ T cells after they are expanded and activated. We demonstrated that engagement of TNFR1 and/or TNFR2 in addition to CD40 prevented CD40 induced proliferation of autoimmune NOD CD4^lo^CD40^+^ T cells [17] as did Fas engagement [13]. However, those treatments did not kill the CD4^lo^CD40^+^ T cells. Because galectin-9 has been shown to induce cell death in Th1 cells [27] we determined whether it could also affect NOD CD4^lo^CD40^+^ T cells. Galectin-9 prevented CD40 induced proliferation in a dose dependent manner (Fig. 1A) and induced necrotic cell death in the CD40 stimulated cells (Fig. 1B; left bar graph). Cells that were isotype treated for four days underwent necrotic cell death and a large portion of that was prevented by CD40-stimulation (Fig. 1B; 49.5% and 26.3% cell death respectively). Addition of a lower concentration, 2.5 µg/ml, of galectin-9 did not prevent the CD40 induced survival (Fig. 1B; 29% cell death), however, when higher concentrations were added much of the CD40 induced survival was prevented. Cell death rates were confirmed with absolute cell counts in trypan blue (data not shown). Apoptotic cell death was not apparent in the CD4^lo^CD40^+^ T cells (Fig. 1B; right bar graph).

In Figure 1B we revealed that galectin-9 added at the time of CD40 engagement caused the cells to die. We then determined if galectin-9, added 24 hours after CD40 engagement, could cause prevention of proliferation and induce cell death despite the fact that at this point survival/proliferation signals and anti-apoptotic protein expression are already in operation [13]. Indeed, when galectin-9 was added to NOD CD4^lo^CD40^+^ T cells 24 hours after CD40 engagement it stopped most of the proliferation and caused death of the cells (Fig. 1C).

Considering that galectin-9 was shown to induce Th1 cell death via ligation of Tim-3 [27], we determined whether galectin-9 suppression of CD40 induced proliferation and galectin-9 induced cell death in the pathogenic NOD CD4^lo^CD40^+^ T cells could be attributed to this pathway. Sorted cells were pretreated, or not, with a Tim-3 blocking antibody then CD40 was stimulated in the absence/presence of galectin-9. Interestingly, both the galectin-9 suppression of CD40 induced proliferation and the induced cell death were completely independent of Tim-3 in this pathogenic CD4 T cell subset (Fig. 1 D and E).

### Galectin-9 exerts its proliferation inhibitory and cell death inducing effects on CD4^lo^CD40^+^ T cells via the carbohydrate recognition domains of the protein

**Figure 2:**
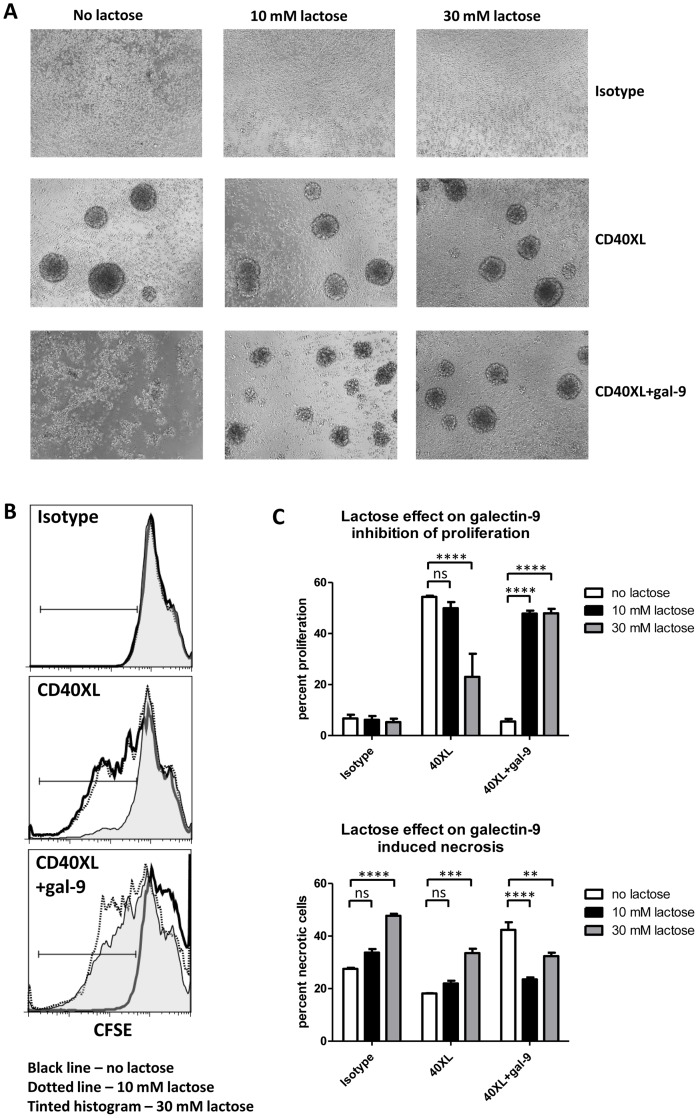
Galectin-9 exerts its proliferation inhibitory and cell death inducing effects on CD4^lo^CD40^+^ T cells via the carbohydrate recognition domains of the protein. CD4^lo^CD40^+^ T cells were sorted from 7–13 week old female NOD spleens and were CFSE labeled. Cells were either isotype treated (Isotype) or CD40 was stimulated (CD40XL) in the absence/presence of 7.5 µg/ml galectin-9 (gal-9) for 4 days. Lactose was added as a competitor for the CRD of galectin-9. (**A**) Cell cluster formation was observed. (**B**) Proliferation was measured by CFSE dilution. (**C**) Bar graphs representing proliferation and necrotic cell death. Asterisks in C denote significant differences determined by one-way Anova; ns – not significant; ** – P between 0.001 and 0.01; *** – P <0.001; **** – P<0.0001. Data represent experiments done on three individual mice of different ages.

Galectin-9 consists of two different carbohydrate recognition domains (CRD) connected to each other with a linker polypeptide chain [24]. The interaction of galectin-9 with its receptors is dependent on the CRD and carbohydrates on the receptor proteins. Therefore we determined whether the galectin-9 effects on CD4^lo^CD40^+^ T cells were preventable by lactose, a competitive substrate that inhibits the interaction of galectins with their receptors. We have demonstrated previously that NOD CD4^lo^CD40^+^ T cells form distinct ball shaped clusters when CD40 engaged [17]. When galectin-9 was added to CD40 engaged NOD CD4^lo^CD40^+^ T cells those clusters no longer formed (Fig. 2A). However, when lactose was added the cluster formation was regained. Similarly, when proliferation was assessed, the galectin-9 inhibition of CD40 induced proliferation was prevented by the addition of lactose (Fig. 2B, bottom histogram and 2C). Interestingly, the higher concentration, 30 mM, of lactose inhibited the CD40 induced proliferation (Fig. 2B, middle histogram and 2C). This could be due to that other galectins may be involved in survival and proliferative responses and would be competed for by lactose as well. When cell death was assessed, 30 mM lactose, but not 10 mM, induced increased necrotic cell death in the isotype treated and CD40 engaged cells (Fig. 2C) again demonstrating that other galectins may be involved in survival. When lactose was added to cells treated with CD40 engagement plus galectin-9, the galectin-9 induced cell death was prevented demonstrating that the CRD of galectin-9 is involved in the mechanism leading to cell death (Fig. 2C).

### *In-vivo* induced CD4^lo^CD40^+^ T cells from BALB/c mice acquire the ability to proliferate in response to CD40 in-vitro and galectin-9 is able to prevent that proliferation

**Figure 3:**
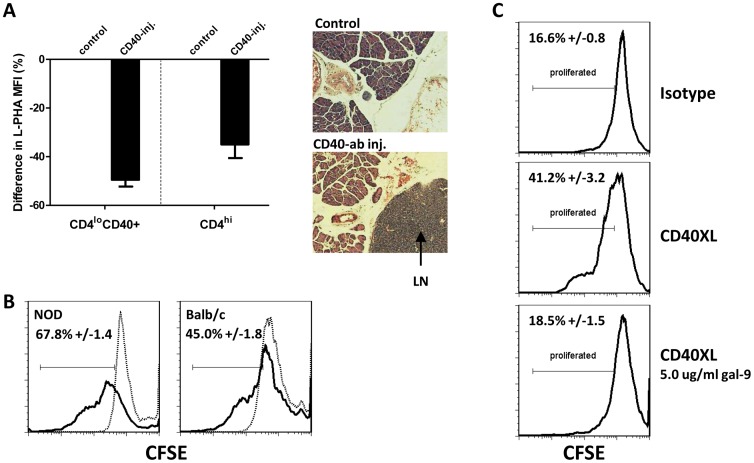
In-vivo CD40 signals cause non-autoimmune CD4^lo^CD40^+^ T cells to appear more like autoimmune CD4^lo^CD40^+^ T cells. (**A**) 10–12 week old, female BALB/c mice were injected i.p. with 1C10+FGK45 agonistic CD40 antibodies (CD40-inj.) or with isotype control (control) antibody. Three days post-injection, splenic CD4^lo^CD40^+^ and CD4^hi^ T cells were purified and stained with L-PHA. Graph depicts the difference in L-PHA stain compared to control. Data are represented as means with SEM. Images are H&E stained sections of pancreata. (**B**) BALB/c mice were treated and CD4^lo^CD40^+^ T cells purified as in A. CD4^lo^CD40^+^ T cells from 10–12 weeks old female NOD spleens were purified for comparison. Cells were CFSE labeled then CD40-stimulated for 4 days. CFSE dilution was measured. Dotted line – isotype treated; solid line – CD40-stimunlated. (**C**) 10 week old female BALB/c mice were injected and CD4^lo^CD40^+^ T cells purified as in A, then cells were labeled with CFSE and isotype treated (Isotype) or CD40 stimulated (CD40XL) in the absence/presence of galectin-9. CFSE dilution was measured. Percentages in B and C are means +/− SEM. Experiments were performed at least three separate times.

We previously demonstrated that injection of non-autoimmune BALB/c mice with agonistic CD40 antibodies expanded the CD4^lo^CD40^+^ T cell population after three to six days to numbers seen in autoimmune NOD mice [17]. Since that treatment also altered the CD40 glycoform profile in those cells we determined whether CD40 signals had an impact on glycosylation status beyond just the CD40 protein itself in CD4 T cells. The extent of β1,6GlcNAc-branched N-glycans was assayed using L-PHA, a plant lectin that binds to carbohydrate structures on glycoproteins that are also recognized by galectins [23]. Injection of agonistic CD40 antibodies into non-autoimmune BALB/c mice caused a reduction in β1,6GlcNAc-branched N-glycans on CD4^lo^CD40^+^ and CD4^hi^ T cells to a level similar to that of autoimmune NOD mice [23] (Fig. 3A; bar graph). This then could have a direct impact on the activation status of those cells as has been shown [23]. After fourteen days, the splenic expansion of CD4^lo^CD40^+^ T cells in the BALB/c mice receded to normal numbers (unpublished data). Interestingly, in response to the *in-vivo* CD40 stimulation, the pancreatic lymph nodes became populated while in control mice no populated nodes were found (Fig. 3A; H&E stained images). CD4^lo^CD40^+^ T cells from control BALB/c mice do not proliferate in response to CD40 stimulation *in-vitro* [13]. We determined whether the expanded CD4^lo^CD40^+^ T cells had acquired the ability to proliferate in response to CD40 *in-vitro* in the same manner as NOD CD4^lo^CD40^+^ T cells do [13] (Fig. 1A). CD4^lo^CD40^+^ T cells purified three days after a single injection with agonistic CD40 antibodies readily proliferated in response to CD40 engagement *in-vitro* (Fig. 3B). However, the expanded BALB/c CD4^lo^CD40^+^ T cells did not proliferate to the same extent as NOD CD4^lo^CD40^+^ T cells; 45% of BALB/c CD4^lo^CD40^+^ T cells proliferated compared to 67.8% of NOD CD4^lo^CD40^+^ T cells (Fig. 3B). As CD4^lo^CD40^+^ T cells from untreated BALB/c mice do not proliferate *in-vitro* in response to CD40 engagement [13] and since expanded BALB/c CD4^lo^CD40^+^ T cells display a CD40 glycoform profile similar to NOD CD4^lo^CD40^+^ T cells [17], the new data indicates that the acquisition of the ‘correct’ CD40 glycoform profile is required before induction of proliferation through CD40 *in-vitro* is possible.

We determined whether addition of galectin-9 could inhibit the CD40 induced proliferation of expanded BALB/c CD4^lo^CD40^+^ T cells. As it did in NOD CD4^lo^CD40^+^ T cells, galectin-9 inhibited the CD40 induced proliferation of the BALB/c CD4^lo^CD40^+^ T cells although a lower concentration was sufficient to achieve complete inhibition of the induced proliferation (Fig. 3C compared to Fig. 1A).

### Galectin-9 interacts with CD40

**Figure 4:**
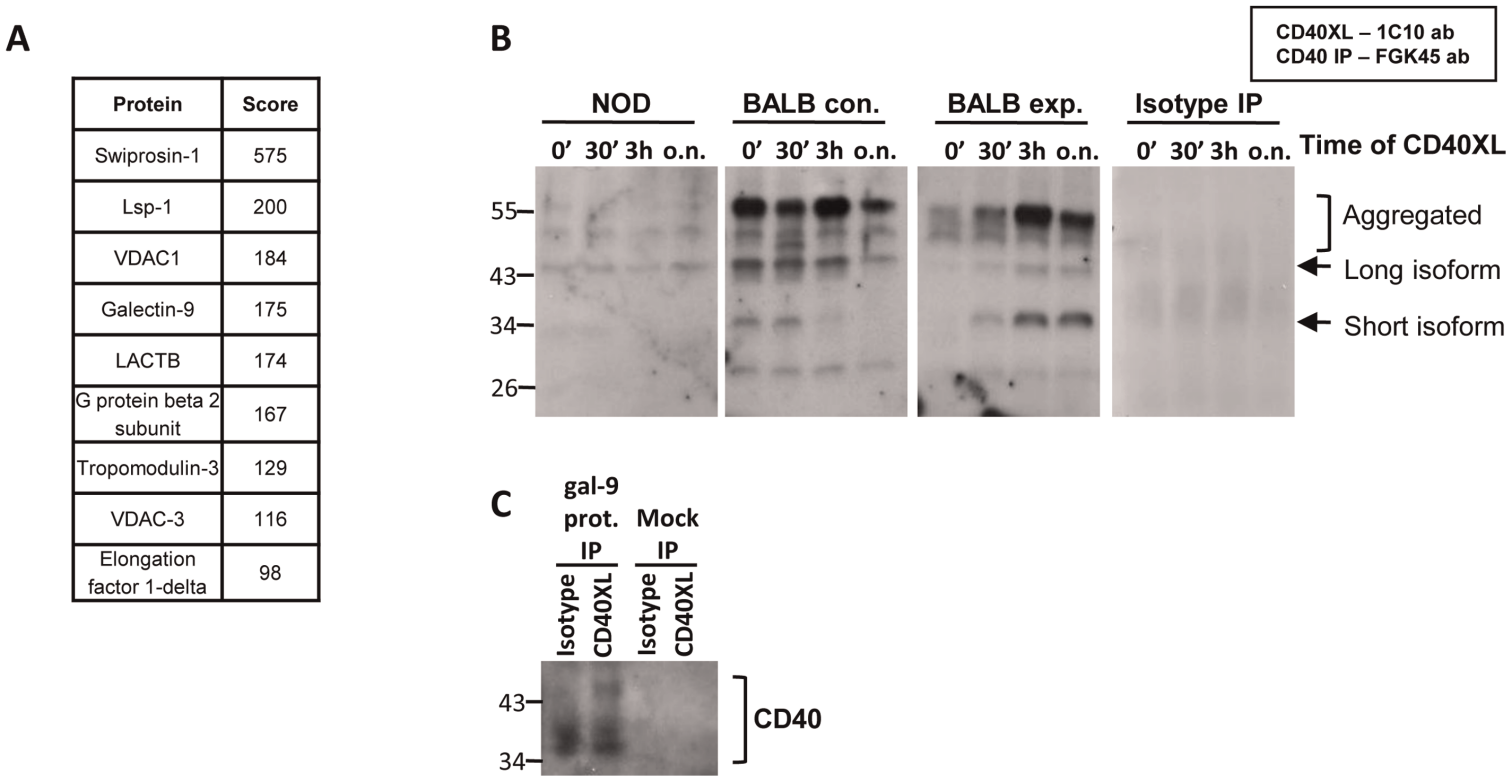
Galectin-9 interacts with CD40. CD4^lo^CD40^+^ T cells were sorted from female NOD, control BALB/c (BALB con.), or agonistic CD40 antibody injected BALB/c (BALB exp.) spleens. Whole cell lysates were prepared. (**A**) CD40 was immunoprecipitated and resulting proteins were separated by SDS-PAGE. Protein bands were sequenced by LC-LC-MS. (**B**) Cells were CD40 stimulated (CD40XL) for 0, 30 minutes, 3 hours or overnight (0′, 30′, 3h, o.n.) then lysates were prepared and CD40 immunoprecipitated. Co-imunoprecipitation of galectin-9 was measured in western blots. Data represents three separate experiments on mice ranging from 8 – 10 weeks old. (**C**) Cells were isotype or CD40 stimulated (CD40XL) overnight then lysates were prepared. Galectin-9 protein conjugated directly to magnetic beads was used in immunoprecipitations. Mock treated beads were used as a control. CD40 was measured in the immunoprecipitated material by western blot. Data represents three separate mice of two different ages (8 and 10 weeks old).

Because galectin-9 had a profound impact on CD40 induced survival and proliferation of CD4^lo^CD40^+^ T cells we determined whether galectin-9 could directly interact with CD40. We performed CD40 co-immunoprecipitation on NOD CD4^lo^CD40^+^ T cell whole cell extracts and separated the resulting proteins on polyacrylamide gels followed by LC-LC-MS identification. Several proteins were identified with galectin-9 being one of them (Fig. 4A). These cells had no exogenously added galectin-9 and therefore the co-immunoprecipitated galectin-9 represents endogenous galectin-9. Other proteins of specific interest are VDAC1 (Voltage-Dependent Anion-selective Channel protein) and VDAC3. CD40 induces high levels of Bcl-X_L_ in NOD CD4^lo^CD40^+^ T cells [13] and VDAC1 and VDAC3 are proteins found in the mitochondrial outer membrane where they interact with proteins such as Bcl-X_L_ to prevent the release of apoptogenic proteins [30]. To corroborate the galectin-9 protein sequencing data we performed CD40 co-immunoprecipitation followed by western blot for galectin-9 on both NOD and BALB/c CD4^lo^CD40^+^ T cells. While there was a weak interaction between CD40 and galectin-9 in NOD CD4^lo^CD40^+^ T cells, that interaction was much stronger in control BALB/c CD4^lo^CD40^+^ T cells (Fig. 4 B). When *in-vivo* expanded BALB/c CD4^lo^CD40^+^ T cells were examined the interaction was less strong at earlier time points of CD40 engagement but became stronger with longer time (Fig. 4B). Although the isotype immunoprecipitation did not yield any galectin-9, since galectins technically could interact with carbohydrates on an antibody we performed co-immunoprecipitations utilizing galectin-9 protein immobilized onto magnetic microbeads. This co-immunoprecipitation strategy revealed CD40 protein in a western blot (Fig. 4C) demonstrating again that the two proteins interact directly.

### Galectin-9 causes up-regulation of TCR associated molecules on the surface of autoimmune CD4^lo^CD40^+^ T cells such that the cells appear similar to conventional CD4^hi^CD3^hi^ T cells

**Figure 5:**
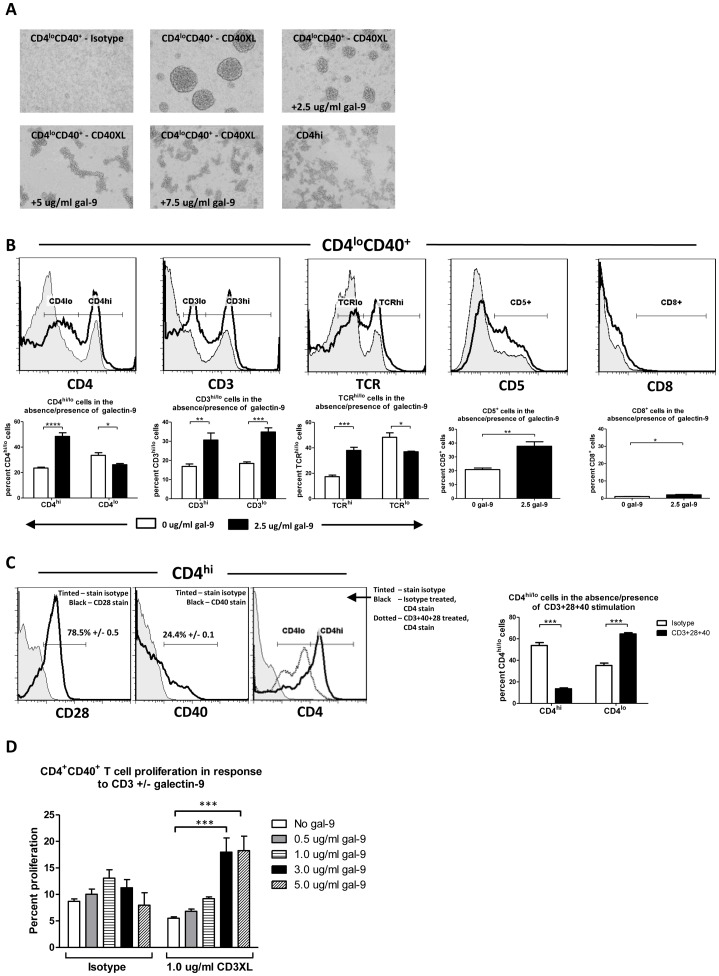
Galectin-9 causes autoimmune CD4^lo^CD40^+^ T cells to appear more like CD4^hi^ T cells and increases CD3 induced proliferation. CD4^lo^CD40^+^ and CD4^hi^ T cells were sorted from 7–13 weeks old female NOD spleens. (**A**) CD4^lo^CD40^+^ T cells were isotype treated (Isotype) or CD40-stimulated for 2 days in the absence/presence of indicated concentrations of galectin-9 then photographed under a microscope. (**B**) NOD CD4^lo^CD40^+^ T cells were treated for 2 hours with 2.5 µg/ml galectin-9 then stained for CD3, CD4, TCR, CD5 and CD8. Tinted – untreated; black line – 2.5 µg/ml galectin-9. Gates were set based on appropriate isotype controls. Below each histogram is a corresponding bar graph depicting the cumulative data. (**C**) NOD CD4^hi^ T cells were either stained immediately for CD28 or CD40 (left two histograms; grey tinted – stain-isotype; black line – CD28 or CD40 as indicated in figure; percentages are means with SEM) or isotype treated or stimulated with CD3+CD28+CD40 overnight then stained for CD4 (bar graph and right histogram; grey tinted – stain-isotype; black line – isotype treated; dotted line – CD3+CD28+CD40 stimulated). (**D**) NOD CD4^lo^CD40^+^ T cells were CFSE labeled then isotype treated (Isotype) or CD3-stimulated (CD3XL) in the absence/presence of indicated concentrations of galectin-9 (gal-9) for 8 days. Proliferation was measured by CFSE dilution. All bar graphs in this figure depict means with SEM. Asterisks denote significant differences determined by one- or two-way Anova as appropriate; ns – not significant; * – P between 0.01 and 0.05; ** – P <0.01; *** – P <0.001; **** – P<0.0001. Experiments were done at least three separate times.

When culturing CD4^lo^CD40^+^ T cells in the presence of galectin-9, a change in the morphology was noted. Normally, when those cells are CD40 engaged distinct ball-shaped clusters are formed [17] while CD4^hi^ T cells form more elongated clusters (Fig. 5A). In the presence of galectin-9 the CD4^lo^CD40^+^ T cells took on a morphology that closely resembled that of CD4^hi^ T cells in culture (Fig. 5A). This led us to determine whether the galectin-9 treated CD4^lo^CD40^+^ T cells also had a protein surface expression resembling that of CD4^hi^ T cells. CD4^lo^CD40^+^ T cells have commonly been discarded or gated out as non-T cells due to their low surface expression of CD4, CD3 and TCR but we demonstrated that those cells have high levels of those proteins intracellularly [13]. NOD CD4^lo^CD40^+^ T cells were treated with sub-lethal levels, 2 5 µg/ml, of galectin-9 then stained for CD4, CD3, TCR and CD5. Those proteins were up-regulated on the treated cells as early as two hours after the treatment (Fig. 5B). A stain for CD8 revealed extremely few, 1–2%, CD8+ cells confirming that the CD4^lo^CD40^+^ T cells conform to the CD4 lineage.

Phorbol-myristate-acetate activation of CD4 T cells is known to down regulate the surface expression of CD4 on those cells [31]. CD3 in combination with costimulus of CD28 is a classic way to activate CD4 T cells. However, recently it was shown that CD40 can serve a costimulatory role in CD4 T cells [32], [33]. Therefore we examined whether CD4^hi^ T cells could be activation induced to appear more like CD4^lo^ cells in terms of CD4 surface expression. We determined that sorted CD4^hi^ T cells from NOD mice were largely CD28^+^ (Fig. 5C) and that although they are largely CD40^−^, a portion of them express low levels of CD40 (Fig. 5C). Interestingly, when CD3 was stimulated together with both CD28 and CD40 the expression of CD4 on the surface of those cells was significantly decreased (Fig. 5C).

### Galectin-9 renders autoimmune CD4^lo^CD40^+^ T cells better able to respond to CD3 stimulation

As demonstrated in Figure 5B, galectin-9 at the lower, sub-lethal concentration, induced higher surface expression of CD3 on autoimmune CD4^lo^CD40^+^ T cells. We therefore determined whether this treatment could cause an increase in response to CD3 stimulation of these cells. Untreated CD4^lo^CD40^+^ T cells spontaneously proliferated over 8 days but when absolute numbers of cells were counted it was revealed that they proliferated and died (Fig. 5D; data not shown). When CD3 was stimulated, the spontaneous proliferation was tempered (Fig. 5D) and with the addition of galectin-9, proliferation was increased in response to CD3 stimulation (Fig. 5D).

**Figure 6:**
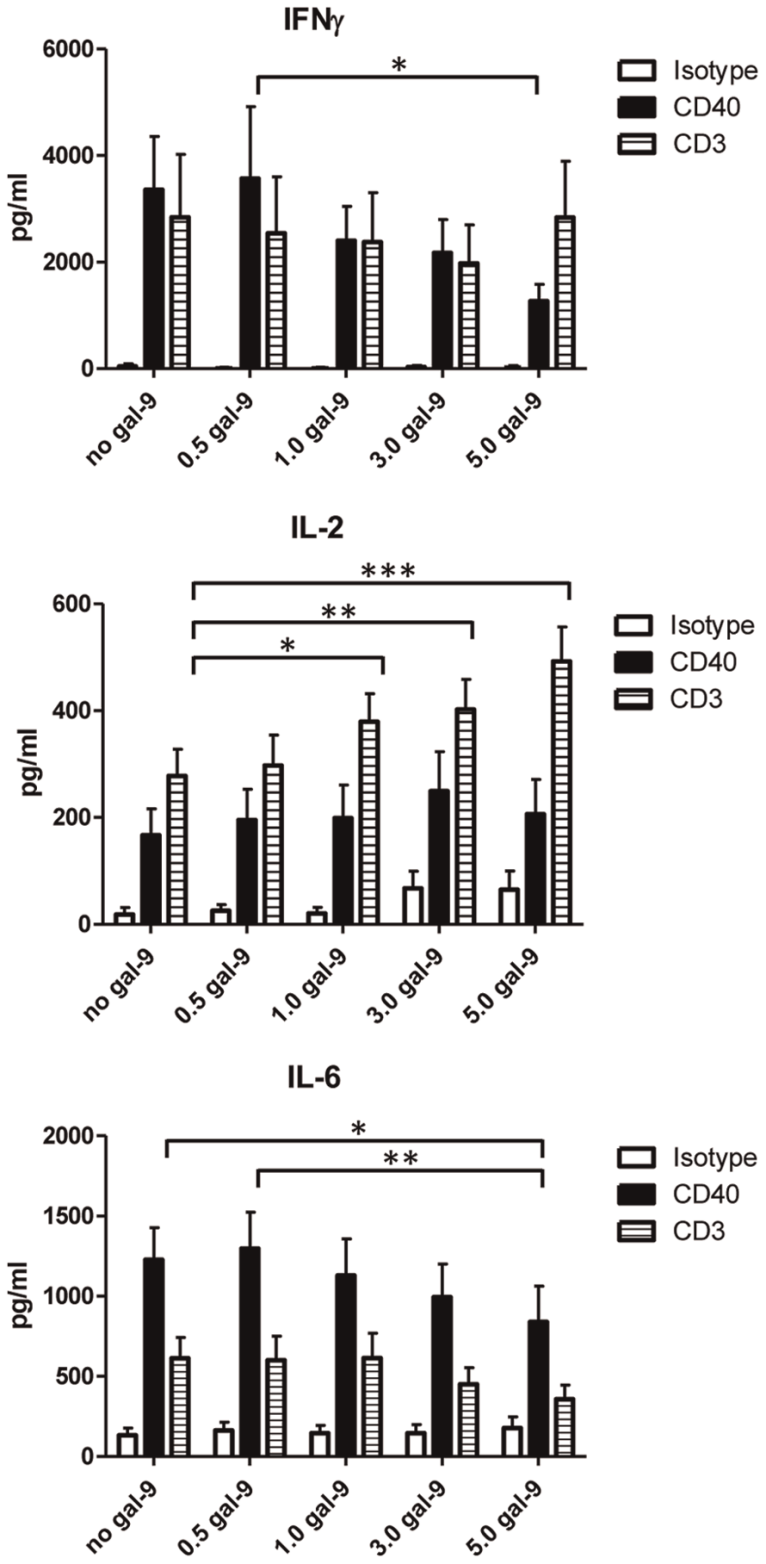
CD40 and CD3 induced cytokine phenotypes differ and galectin-9 alters the production level. CD4^lo^CD40^+^ T cells were sorted from 7–20 weeks old female NOD spleens. Cells were either isotype treated, CD40- or CD3-stimulated in the absence/presence of indicated concentrations of galectin-9 (gal-9; µg/ml) for 3 days then cytokines were measured. Bar graphs depict means with SEM. Asterisks denote significant differences determined by one-way Anova; * – P between 0.01 and 0.05; ** – P <0.01; *** – P <0.001. Measurements were done on four individual mice of different ages.

Considering the impact of galectin-9 on proliferative responses of CD4^lo^CD40^+^ T cells to CD3-stimulation we determined whether galectin-9 could also impact the production of cytokines by these cells. We determined the level of production of IL-2, IL-4, IL-5, IL-6, IL-10, IL-17, and IFNγ. Regardless of the presence of galectin-9, the cytokine production was very different depending on whether CD40 or CD3 was stimulated (Fig. 6 and S1). CD40 induced more IL-6 (Fig. 6) and IL-10 (Fig. S1) while CD3 induced more IL-17 (more than double the amount compared to CD40; Fig. S1) and IL-2 (Fig. 6) after 3 days of stimulation. IFNγ production was similar between the two treatments (Fig. 6). CD40-induced IFNγ and IL-6 production was affected by the addition of galectin-9 where production was down-regulated in a dose dependent manner (IFNγ, P<0.05; IL-6, P<0.01). Conversely, CD3 induced IL-2 production was increased by the addition of galectin-9 (P<0.001). CD40 did not induce any IL-4 or IL-5 in these cells and while CD3 induced some, the levels were very low (Fig. S1).

### Expression of some cytokines is enhanced when both CD3 and CD40 are engaged

**Figure 7:**
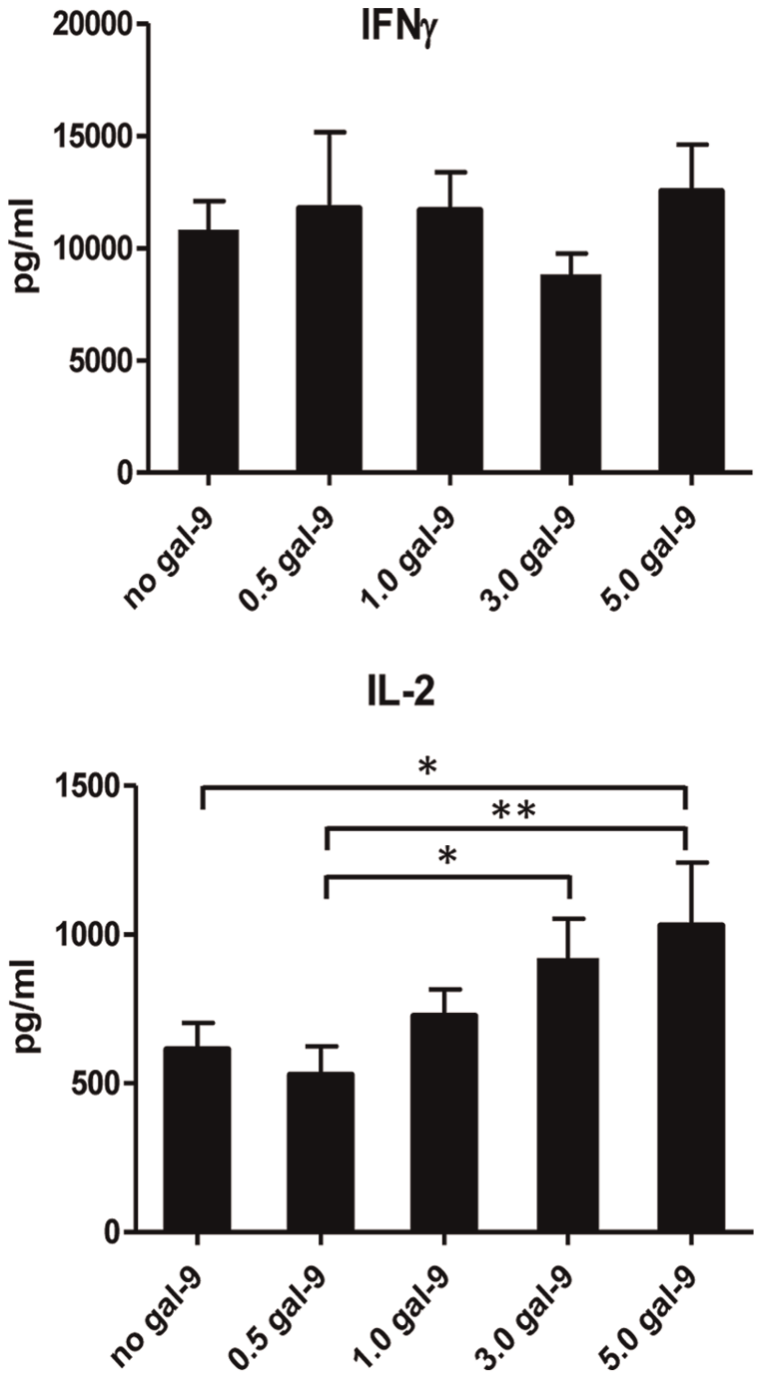
CD3 and CD40 co-stimulation increases production of some cytokines. Data represented in this figure were part of experiments done in Figure 6 and can therefore be directly compared to Figure 6. Cells were sorted as in Figure 6 and were CD3- and CD40-stimulated in the absence/presence of indicated concentrations of galectin-9 (gal-9; µg/ml) for 3 days then cytokines were measured. Bar graphs depict means with SEM. Asterisks denote significant differences determined by one-way Anova; * – P between 0.01 and 0.05; ** – P <0.01. Measurements were done on four individual mice of different ages.

CD40 has been demonstrated to be a co-stimulatory molecule on T cells, comparable in its function to CD28 [32]. Therefore we determined the cytokine production by NOD CD4^lo^CD40^+^ T cells in response to simultaneous CD3 and CD40 stimulation. IFNγ and IL-2 were induced to significantly higher levels during co-stimulation compared to single treatment (Fig. 7; note scale changes compared to Fig. 6). The other cytokine levels were unaffected by the co-stimulation. Interestingly, while IFNγ production more than doubled, galectin-9 addition no longer impacted the level of production. IL-2 production was doubled by the co-stimulation and remained sensitive to galectin-9 which increased the induced levels (Fig. 7; note scale changes compared to Fig. 6).

## Discussion

CD40 is known to play a pivotal role in autoimmune disease and has gained interest as a target in transplantation and cancer therapies. In autoimmunity the major focus has been on signaling outcomes, associated signaling molecules, and on preventing its ligand, CD154, from signaling through CD40. However, little work has been done focusing on the actual CD40 receptor itself and how to control it. We recently demonstrated that CD40 is not one but several dynamic receptors composed of different glycoforms of CD40 isoform I and that a less glycosylated form is associated with survival and proliferation signals [17]. Interestingly, when the receptor(s) is engaged, hybrid receptors can form with TNFR1 and TNFR2 [17]. Here we reveal a role for CD40 that could have far-reaching impact on the status of the immune system. *In-vivo*, CD40 signaling appears to access glycosylation events to result in a decrease in β1,6GlcNAc-branched N-glycans in the CD4 T cell compartment. This could directly affect how galectins interact with surface glycoproteins and therefore cause a lowered threshold of activation [23]. Presumably, a network of cell types in addition to CD4 T cells is involved in this *in-vivo* CD40-induced alteration in glycosylation as we were not able to induce the same change in response to CD40 *in-vitro* with purified BALB/c CD4^lo^CD40^+^ T cells (data not shown). If that is the case this effect on glycosylation events could explain why CD40 appears to have a vast influence on the immune system as a whole regardless of cell type.

Controlling CD40 signaling has long been an elusive goal in autoimmunity. All known antibodies to CD40 appear to be agonistic in nature and blocking its ligand, CD154, is only successful when administered prior to activation of autoimmune processes [14], [18]. Here we demonstrate that galectin-9 is able to control culprit CD4^lo^CD40^+^ T cells in T1D not only prior to CD40 engagement but also after it has induced survival/proliferation signals. We also demonstrate that higher concentrations of galectin-9 induce cell death in NOD CD4^lo^CD40^+^ T cells, suggesting a mechanism to control these pathogenic cells in autoimmunity. Interestingly, that mechanism is independent of Tim-3 which has been shown to be involved in galectin-9 induced death of Th1 cells [27]. Instead galectin-9 apparently exerts its effect on CD4^lo^CD40^+^ T cells via direct interaction with CD40.

Injection of non-autoimmune BALB/c mice with agonistic CD40 antibodies to expand the CD4^lo^CD40^+^ T cell population changed not only the CD40 glycoform profile [17] but also the glycosylation status of those cells to levels seen in autoimmune NOD mice [23]. This also disrupted the galectin-9/CD40 interaction. However, this disruption was evident only at the early timepoints immediately *ex-vivo* and the interaction then started to recover at the later timepoints suggesting a reason for why the *in-vitro* CD40 induced proliferation of those expanded cells was not as profound as in the same population from NOD mice. Therefore, galectin-9 control of CD40 signals in CD4^lo^CD40^+^ T cells could be one mode of action for galectin-9 to be protective against diabetes as was demonstrated [28]. However, as several other cells are affected by galectin-9, including Th1 cells [27], [28], galectin-9 inhibition of T1D must include several modalities.

Stimulation through CD40 has been utilized as an adjuvant in vaccines [34], [35], [36]. Therefore our data also indicate that CD4^lo^CD40^+^ T cells take part in normal immune responses since agonistic antibody injection of non-autoimmune BALB/c mice expanded that cell subset. After fourteen days the CD40 induced expansion of CD4^lo^CD40^+^ T cells in BALB/c mice receded to normal numbers (data not shown). Given the persistence of CD40 signals in autoimmunity [13] this then suggests that in the autoimmune setting, mechanisms to control CD4^lo^CD40^+^ T cells are unavailable or are thwarted by persistent CD40 signals such that CD4^lo^CD40^+^ T cell expansions, that may have started as part of a normal immune response, are maintained rather than allowed to recede.

A very interesting finding is that galectin-9, at sub-lethal concentrations, causes increased surface expression of CD4, CD3, TCR and CD5 on NOD CD4^lo^CD40^+^ T cells, essentially de-cloaking these cells that have long been overlooked precisely because of their low surface expression of those defining molecules. The fact that CD4^hi^ T cells can be activation induced by CD3+CD28+CD40 stimulation to express lower levels of CD4 on their surface underscores that CD4 T cells are dynamic. Functionality of the autoimmune CD4^lo^CD40^+^ T cells is affected by the availability of CD3 which in turn is affected by galectin-9. The very different CD4^lo^CD40^+^ T cell cytokine production in response to CD3 compared to CD40 and the modulation of the levels produced in the absence/presence of galectin-9 supports this scenario. Interestingly, CD40 stimulation favors production of IL-6 and CD3 stimulation favors production of IL-17 and IL-2 while IFNγ is induced to similar levels in response to either treatment. This suggests that, depending on the microenvironment and on the availability of signaling molecules on the surface of the cell, an effector cell can respond very differently in terms of cytokine output and in terms of whether it survives/proliferates or not. Therefore it appears that different Th1 phenotypes, including one that can simultaneously express IFNγ and IL-17, can exist in the same cell population depending on the surface availability and engagement of the CD40 and CD3 molecules. In addition, the CD3-induced increase in IL-2 production in the presence of galectin-9 could mean that if a CD4^lo^CD40^+^ T cell is able to be stimulated through CD3 instead of CD40 it would be able to support the function of regulatory T cells which are known to be dependent on IL-2 [37]. However, the levels of IFNγ and IL-2 production were affected by CD3 and CD40 co-stimulation which further complicates this issue. The expansion of CD4^lo^CD40^+^ T cells in autoimmunity [12], [14], [15], [16], their often low surface expression of CD3[14], [38] and the constant signaling through CD40 on those cells [13] indicates that autoimmune mouse CD4^lo^CD40^+^ T cells may not be able to readily receive CD3 signals. This then would perpetuate the CD40 response and the CD40 driven cytokine phenotype.

Galectin-9 is known to suppress IL-17 production [39], [40]. Contrary to those results, in this manuscript galectin-9 had no effect on IL-17 production by CD4^lo^CD40^+^ T cells. It is possible that the different disease models are the reason for this discrepancy, however, more likely is that the discrepancy is due to that different cell subsets were studied. CD4^lo^CD40^+^ T cells studied here are typically gated out by other investigators due to their low surface expression of CD4. Therefore a direct comparison of IL-17 levels with other publications may be inappropriate.

**Figure 8:**
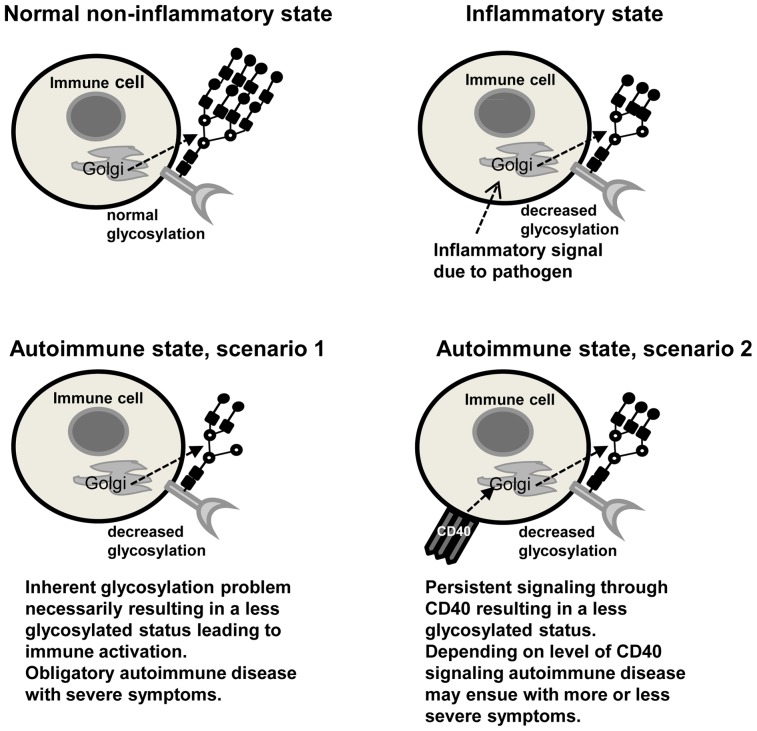
Glycosylation and immune status. Cartoon depicting different possibilities for immune activation during normal and disease states.

The data presented in this manuscript point to a new area of exploration in controlling autoimmunity. It is possible that certain defects in the glycosylation machinery would obligatorily lead to autoimmunity but in a situation where glycosylation processes are intact, excessive CD40 signaling could cause immune activating changes in glycosylation status and then lead to autoimmunity (Fig. 8). Depending on how persistent and extensive the CD40 signaling is, different gradations of immune activation could occur. This scenario could explain differences in time of onset and severity within a given autoimmune disease.

## Materials and Methods

### Mice

NOD and BALB/c mice were from Taconic (Hudson, NY, USA) and Jackson Laboratories (Bar Harbor, ME, USA) respectively, and were housed under pathogen free conditions at the University of Colorado Denver, AAALAC-approved facility and all experiments were carried out under IACUC-approved protocol number 55809(03)1E. The NOD mice consistently achieve >90% diabetes in females by the age of 18 weeks.

### Ethics statement

This study was carried out in strict accordance with the recommendations in the Guide for the Care and Use of Laboratory Animals of the National Institutes of Health. All experiments conducted in this study were carried out under University of Colorado Denver Institutional Animal Care and Use Committee approved protocol number 55809(03)1E.

### Antibodies and reagents

MHCII-, CD8- and CD4-conjugated microbeads for cell-sorting were purchased from Miltenyi Biotec (Auburn, CA, USA). CD40 antibodies 1C10 [41] and FGK45 [42] were produced and biotin or FITC conjugated in house. Isotype antibodies, Tim-3 antibody (8B.2C12), biotin-conjugated CD3 (145–2C11) and CD28 (37.51) antibodies, fluorochrome conjugated antibodies to CD3 (145.2C11; PE), TCR (H57–597; PE–Cy5), CD5 (53–7.3; FITC), CD8 (53–6.7; PE), and CD28 (37.51; PE) were purchased from eBioscience, Inc. (San Diego, CA, USA). Anti-CD4 (H129.19; PE-Cy5) was from BD Pharmingen (San Diego, CA, USA). P. vulgaris Leukoagglutinin (L-PHA) was from Vector Laboratories (Burlingame, CA, USA). Recombinant human galectin-9 (which shares 70% amino acid sequence identity with mouse galectin-9) was purchased from R&D Systems® (Minneapolis, MN, USA). DMEM was from HyClone Laboratories, Inc. (Logan, UT, USA) and fetal calf serum from Gemini Bio Products (Atlanta, GA, USA). Magnetic beads used in immunoprecipitations, MyOne™ tosylactivated Dynabeads®, were from Invitrogen (Grand Island, NY, USA). Cytokine analysis kit was from eBioscience, Inc. (San Diego, CA, USA). All other reagents were from Sigma-Aldrich® (St. Louis, MO, USA).

### T cell purification and cell culture

Splenic CD4^lo^CD40^+^ and CD4^hi^ T cells from 7 – 13 week old, female NOD or BALB/c mice were sorted using an autoMACS™ (Miltenyi Biotec, Auburn, CA, USA) as previously described [13] with the exception that directly conjugated CD4-microbeads were used instead of biotinylated CD4-antibody followed by streptavidin-microbeads. This sort results in >90% pure CD4^lo^CD40^+^ cells and >95% pure CD4^hi^ cells. Cells were cultured in DMEM containing 10% fetal calf serum and 50 μM β-mercaptoethanol. Cells were seeded at either 2×10^6^ cells per well, in 0.5 ml medium, in 48-well plates or at 1×10^6^ cells per well, in 0.1 ml medium, in 96-well plates. Cells were CD40 crosslinked using 10 μg/ml of biotinylated anti-CD40 antibody (1C10) or CD28 crosslinked using 5 ug/ml anti-CD28 antibody (37.51) followed by 1 µg/ml of streptavidin. For CD3 crosslinking, 1.0 µg/ml biotinylated anti-CD3 antibody (145-2C11) was used followed by 0.1 µg/ml streptavidin. Experiments using galectin-9 were done at concentrations/times indicated in the figure legends.

### *In-vivo* antibody treatments

To stimulate CD40 *in-vivo*, 10 – 12 week old female BALB/c mice were injected intraperitoneally with 25 µg each of anti-CD40 antibodies 1C10 and FGK45. Three days after the injection, spleens were harvested and cells purified as described above.

### T cell proliferation and death assays

T cell proliferation was measured by CFSE dilution [13]. Apoptotic death rates were measured by intracellular propidium iodide staining as described [43]. Briefly, cells were fixed in paraformaldehyde/PBS then permeabilized in saponin/PBS. Propidium iodide (PI) was added to the fixed/permeabilized cells and allowed to intercalate with the DNA. Apoptotic cells are considered those that stain poorly with PI due to the small DNA size of apoptotic cells. For necrotic death, samples were stained immediately with PI for 10 minutes, without first fixing and permeabilizing the cells, to measure any uptake of propidium iodide due to leaky or damaged cells. In this strategy any strong staining cell, where PI intercalates with the still intact DNA, is considered necrotic. The measurements were confirmed by counting absolute numbers of live versus dead cells in trypan blue.

### Immunoprecipitations and western blots

Sorted and treated cells were lysed in lysis buffer (20 mM Tris-HCl pH 7.5, 2 mM EDTA, 137 mM NaCl, 0.5% Triton X-100, 1 µg/ml each of leupeptin and aprotinin, 0.2 mM PMSF and 0.4 mM sodium-orthovanadate) and incubated for 10 minutes at room temperature then lysates were clarified by centrifuging at 16 K×g for 3 minutes. FGK45-conjugated or galectin-9 protein conjugated magnetic beads (conjugated according to manufacturer's protocol) were immediately added to the lysates and incubated for 20–30 minutes, rotating at room temperature. A strong magnet (Invitrogen) was used to wash the beads three times with lysis buffer then the immunoprecipitated material was eluted using 0.1 M sodium citrate at pH 3.0.

Western blots were performed as described [13].

### Protein sequencing

Protein sequencing was done, by applying LC-LC-MS to protein bands isolated from polyacrylamide gel separated CD40 immunoprecipitates, at the University of Colorado Cancer Center Proteomics Core (University of Colorado Denver, Anschutz Medical Campus; supported by CTSA grant UL1 RR025780 and University of Colorado Comprehensive Cancer Center Core Support P20 CA046934–17).

### Cytokine analysis

Cells were disrupted by freezing them in the growth medium then thawing. Debris was pelleted by centrifugation then cytokines were measured in the supernatant. Cytokine analysis was done using a mouse Th1/Th2 10plex kit and analysis of cytokine levels was done using the software provided with the kit.

### Flow cytometry

Flow cytometry was done on a FACS Calibur flow cytometer (BD Biosciences, San Jose, CA, USA) and analysis performed using FlowJo (Ashland, OR, USA) analysis software.

### Data analysis

Data analysis was performed using GraphPad Prism 5 from GraphPad Software, Inc. (La Jolla, CA, USA). One- or two-way Anova was used, as appropriate, to determine significance.

## Supporting Information

Figure S1 **CD40 and CD3 induced cytokine phenotypes differ.** CD4^lo^CD40^+^ T cells were sorted from 7–20 weeks old female NOD spleens. Cells were either CD40- or CD3-stimulated in the absence/presence of indicated concentrations of galectin-9 (gal-9; µg/ml) for 3 days then cytokines were measured. Bar graphs depict means with SEM. Measurements were done on four individual mice of different ages.
(PDF)
