# Supplementary material for: Galectin-9 Controls CD40 Signaling through a Tim-3 Independent Mechanism and Redirects the Cytokine Profile of Pathogenic T Cells in Autoimmunity
Source: PLoS One. 2012 Jun 7;7(6):e38708. doi: 10.1371/journal.pone.0038708 (PMC3369903; doi:10.1371/journal.pone.0038708)
Supplement: Figure S1 — CD40 and CD3 induced cytokine phenotypes differ. CD4loCD40+ T cells were sorted from 7–20 weeks old female NOD spleens. Cells were either CD40- or CD3-stimulated in the absence/presence of indicated concentrations of galectin-9 (gal-9; µg/ml) for 3 days then cytokines were measured. Bar graphs depict means with SEM. Measurements were done on four individual mice of different ages. (PDF) [file pone.0038708.s001.pdf]

Figure S1.

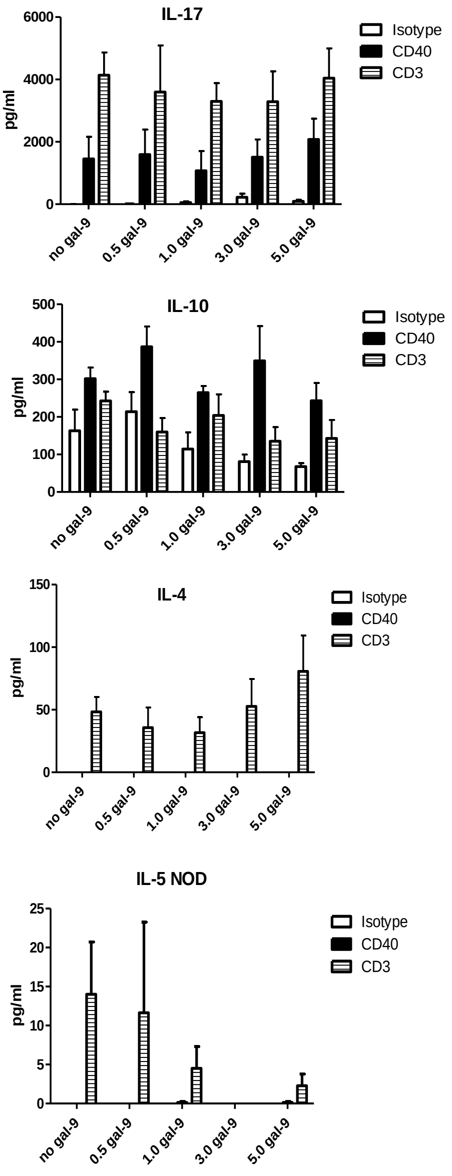

Figure S1. CD40 and CD3 induced cytokine phenotypes differ. $CD4^{lo}CD40^{+}$ T cells were sorted from 7-20 weeks old female NOD spleens. Cells were either CD40- or CD3-stimulated in the absence/presence of indicated concentrations of galectin-9 (gal-9; ug/ml) for 3 days then cytokines were measured. Bar graphs depict means with SEM. Measurements were done on four individual mice of different ages.
